# Serum insulin levels are associated with vulnerable plaque components in the carotid artery: the Rotterdam Study

**DOI:** 10.1530/EJE-19-0620

**Published:** 2020-01-20

**Authors:** Blerim Mujaj, Daniel Bos, Maryam Kavousi, Aad van der Lugt, Jan A Staessen, Oscar H Franco, Meike W Vernooij

**Affiliations:** 1 Departments of Epidemiology, Erasmus MC, Rotterdam, The Netherlands; 2 Radiology and Nuclear Medicine, Erasmus MC, Rotterdam, The Netherlands; 3 Department of Cardiovascular Sciences, Studies Coordinating Centre, Research Unit Hypertension and Cardiovascular Epidemiology, University of Leuven, Leuven, Belgium; 4 Department of Clinical Epidemiology, Harvard TH Chan School of Public Health, Boston, Massachusetts, USA; 5 Cardiovascular Research Institute Maastricht (CARIM), Maastricht University, Maastricht, The Netherlands; 6 Institute of Social and Preventive Medicine (ISPM), University of Bern, Bern, Switzerland

## Abstract

**Background:**

To investigate the association between fasting serum insulin and glucose levels with atherosclerotic plaque composition in the carotid artery. Impaired insulin and glucose levels are implicated in the etiology of cardiovascular disease; however, their influence on the formation and composition of atherosclerotic plaque remains unclear.

**Methods:**

In 1740 participants (mean age 72.9 years, 46% women, 14.4% diabetes mellitus) from the population-based Rotterdam Study, we performed carotid MRI to evaluate the presence of calcification, lipid core, and intraplaque hemorrhage in carotid atherosclerosis. All participants also underwent blood sampling to obtain information on serum insulin and glucose levels. Using logistic regression models, we assessed the association of serum insulin and glucose levels (per s.d. and in tertiles) with the different plaque components, while adjusting for sex, age, intima-media thickness, and cardiovascular risk factors.

**Results:**

Serum insulin levels were associated with the presence of intraplaque hemorrhage (adjusted odds ratio (OR): 1.42 (95% CI: 1.12–1.7)) We found no association with the presence of calcification or lipid core. Sensitivity analyses restricted to individuals without diabetes mellitus yielded similar results. No associations were found between serum glucose levels and any of the plaque components.

**Conclusions:**

Serum insulin levels are associated with the presence of vulnerable components of carotid plaque, specifically with intraplaque hemorrhage. These findings suggest a complex role for serum insulin in the pathophysiology of carotid atherosclerosis and in plaque vulnerability.

## Introduction

Dysregulations in insulin and glucose metabolism, the pathophysiological underpinnings of diabetes mellitus, are associated with an increased risk of cardiovascular disease due to the accelerated accumulation of atherosclerosis (1, 2). Despite abundant evidence for the role of diabetes in the pathophysiology of atherosclerosis and clinical cardiovascular events, insights into the contribution of early disruptions in serum levels of insulin and glucose on the development of atherosclerosis remain scarce. Moreover, levels of serum insulin and their atherogenic properties are even conflicting (3, 4, 5).

Another important topic of interest within the field of atherosclerosis, for which the role of serum levels of insulin and glucose are even more elusive, pertains to plaque composition. Plaque composition is directly related to the chances of a plaque to rupture and potentially result in clinical cardiovascular events (6, 7, 9). The vulnerability of a plaque to rupture is assessed by evaluation of the presence of vulnerable, non-calcified plaque components such as lipid core or intraplaque hemorrhage (9) and the presence of calcification, which is regarded as a more plaque-stabilizing component (3, 4, 5). *In-vivo* visualization of the atherosclerotic plaque and its components can be non-invasively accomplished by MRI (10).

Against this background, we investigated the association between insulin and glucose levels with atherosclerotic plaque composition in the carotid artery in a large population-based cohort of subjects with subclinical atherosclerosis.

## Methods

### Study population

**Figure 1:**
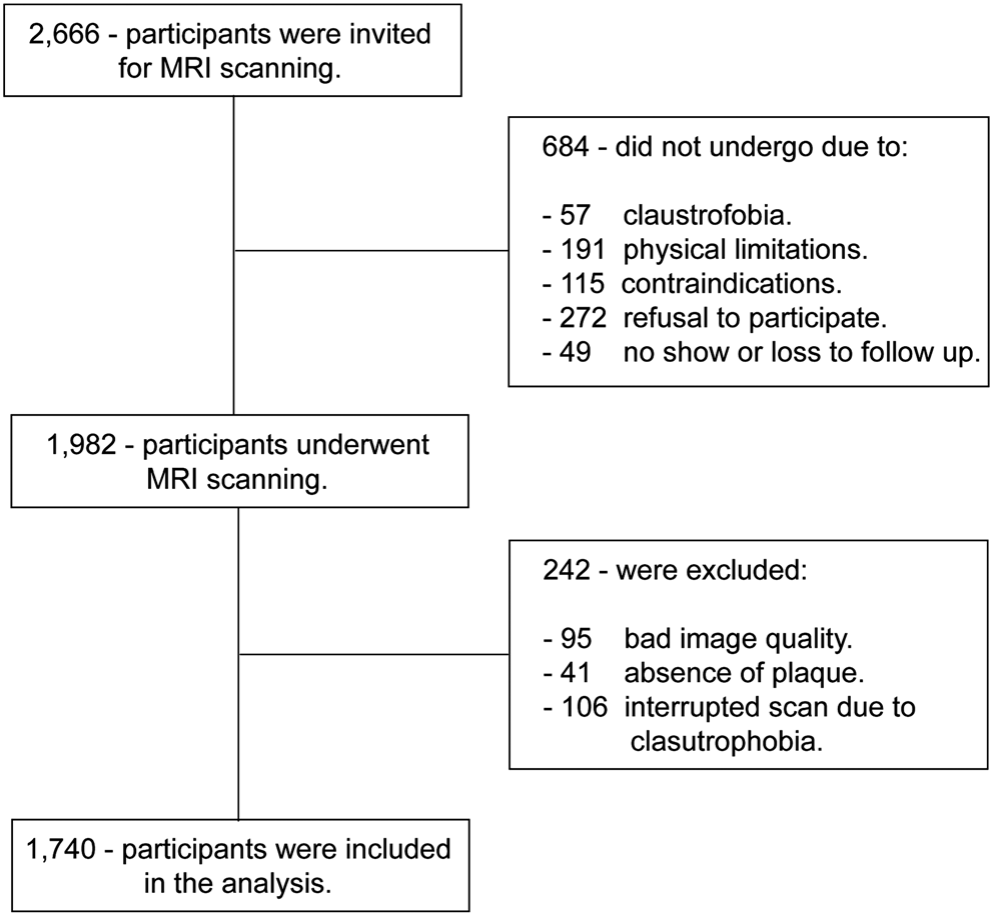
Flow chart of the participants included in the analysis.

The Rotterdam Study is a prospective population-based cohort (11). Between 2007 and 2012, participants with carotid atherosclerosis were invited to undergo an MRI scan of the carotid arteries. Participants were selected for MRI based on the results of carotid artery ultrasound examination (intima-media thickness ≥2.5 mm in one or both carotid arteries) performed in all participants of the Rotterdam Study. From the 2666 invited participants, 272 refused to participate and another 363 did not undergo an MRI scan due to claustrophobia (*n* = 57), physical limitations (*n* = 191), and MRI contraindication (*n* = 115). From the remaining 1982 participants that underwent MRI scan, 242 were excluded due to bad image quality (*n* = 95), the absence of plaque (*n* = 41), or incomplete examinations due to claustrophobia during scanning (*n* = 106). Hence, 1740 participants were included in the analyses (Fig. 1). The Rotterdam Study complies with the Helsinki Declaration and has been approved by the Medical Ethics Committee of the Erasmus MC and by the Dutch Ministry of Health, Welfare, and Sports, implementing the ‘Wet Bevolkings Onderzoek: ERGO (Population Screening Act: Rotterdam Study)’. All participants provided written informed consent to participate in the study and to obtain information from their treating physicians.

### Carotid scanning and analysis of plaque components

A magnetic resonance 1.5 Tesla scanner (GE Healthcare) with a dedicated bilateral phased-array surface coil (Machnet, Eelde, the Netherlands) was used to perform bilateral multisequence imaging of the carotid arteries, with a standardized scanning protocol that required an approximate total scanning time of 30 min. Details of the scanning protocol, reading procedure, and reproducibility are described in detail elsewhere (12, 13). Two independent readers, with 3 years of experience visually evaluated the carotid artery images for the presence of three plaque components, namely intraplaque hemorrhage (IPH), lipid core, and calcification. IPH was defined as the presence of a hyperintense region in the atherosclerotic plaque on 3D-T1w-GRE. Lipid core presence was defined as a hypointense region, not classified as IPH or calcification, in the plaque on PDw-FSE or PDw-EPI and T2w-EPI images or a region of relative signal intensity drop in the T2w-EPI images compared with the PDw-EPI images. Calcification was defined as the presence of a hypointense region in the plaque on all sequences (14). Subjects were recorded as positive for the presence of any plaque component if the component was identified in one or both carotid arteries. To assess the intra-scan variability, 40 participants underwent a second MRI scan (average time between scans 15 ± 9 days). For interobserver reproducibility analyses, random MRI examinations were selected (*n* = 50) and read by a second observer. Intra-scan and interobserver agreement were calculated by using Cohens’ Kappa statistics. The intra-scan agreement was good for all measurements. The Kappa value for the presence of IPH was 0.95 (95% CI: 0.88–0.99), for lipid core 0.85 (95% CI: 0.74–0.96), and for calcification 0.91 (95% CI: 0.82–0.99). The interobserver agreement was good for all measurements. The Kappa value for IPH was 0.86 (95% CI: 0.72–0.99), for lipid core 0.86 (95% CI: 0.72–0.99), and for calcification 0.94 (95% CI: 0.86–0.99) (13).

### Assessment of fasting insulin and glucose levels

The venous blood samples were taken after overnight fasting from all participants at the research center and stored at −80°C in a number of 5-mL aliquots. Serum fasting glucose levels were determined by using the glucose hexokinase method within 1 week after sampling (15). Serum fasting insulin level was determined in samples that had been kept frozen and were measured on a Roche Modular Analytics E170 analyzer (Roche Diagnostics GmbH, Mannheim, Germany) by electrochemiluminescence immunoassay technology. This assay does not cross-react with proinsulin or C-peptide. The intraassay repeatability showed a coefficient of variation of 1.0%. The day-to-day variation of the assay (i.e. intermediate precision) yielded a coefficient of variation of 3.6%. These numbers indicate the excellent reliability of the insulin assay in our study (16). We used the following formula to calculate the HOMA–IR index (fasting insulin (mU/L) × fasting glucose (mmol/L))/22.5 (17). The blood measurements were made for all participants at study entry, and the mean time interval between blood measurements and carotid MRI scan was 7.9 years (s.d. of 4.0 years).

### Other risk factors in the Rotterdam Study

The information about other cardiovascular risk factors as relevant covariables was obtained by interview, physical examination, and blood sampling between the years 1998 and 2008 (11). Diabetes mellitus was defined as fasting blood glucose >6.9 mmol/L, nonfasting glucose >11.0 mmol/L, or use of glucose-lowering medication. Systolic and diastolic blood pressure was measured using a random-zero sphygmomanometer on the right arm and two measurements were averaged for the analysis. Smoking status was assessed by interview and categorized into never, past, and current smoking. BMI was calculated based on the weight in kilograms divided by height in meters squared. Waist circumference (WC) was measured at the midpoint between the lower rib and the iliac crest in all participants with standing position and gentle breathing. Total cholesterol and high-density lipoproteins (HDL) levels were measured using standard laboratory techniques. The information on the use of antihypertensive medication and lipid-lowering medication was obtained from pharmacy records (11). History of stroke or coronary heart disease (CHD) was self-reported at study entry and verified by clinical data from the medical records or the occurrence during study follow-up but before the time of carotid MRI scanning.

### Statistical analysis

The distribution of continuous and categorical variables was described using means (s.d.), medians (interquartile ranges (IQRs)), or percentages. We performed a natural logarithmic transformation to normalize the distributions of serum insulin and glucose. To investigate the association between fasting insulin and glucose levels with intraplaque hemorrhage (IPH), lipid core, and calcification, a three-step statistical analysis approach was used. First, we investigated the association between fasting insulin and glucose levels with the presence of each component in one or both carotid arteries using logistic regression models. In model 1, adjusted for sex, age, intima-media thickness, and the time difference between insulin and glucose measurements and MRI scan. In model 2, additionally adjusted for smoking, serum high-density lipoprotein, serum total cholesterol, systolic and diastolic blood pressure, diabetes mellitus, BMI, waist circumference, use of anti-diabetic medication, use of antihypertensive medication, and insulin or glucose levels, dependent on the determinant under investigation. In model 3, additionally adjusted for the use of lipid-lowering medication (13), vitamin K antagonists and antiplatelet agents (18). Second, we categorized serum insulin and glucose levels into tertiles and investigated the association of tertiles of insulin and glucose (lowest tertile was used as the reference category) with carotid plaque components using regression model 1. Third, we performed the following three sensitivity analyses. In the first analysis, we investigated all the previously mentioned associations only in participants that had their MRI-scan and blood measurements within 1 year in order to assess the potential effect of the time delay between the measurements. In the second analysis, we reassessed all associations in participants that were free of diabetes mellitus at the time of the MRI. In the third analysis, we stratified all analyses for sex, to investigate whether associations are different between males and females. Additionally, we investigated the association between serum insulin and glucose and intima-media thickness and association between HOMA index and carotid composition using regression models. All analyses were carried out using IBM SPSS Statistical package version 21.

## Results

Table 1 shows the population characteristics at the MRI scan. The mean age of the population was 72.9 years (9.1 years) and 46.0% were women. A total of 251 (14.4%) participants were diagnosed with diabetes mellitus at baseline. The median (IQR) fasting insulin level was 74 (50–98) pmol/L and the median (IQR) fasting glucose level was 5.6 (5.2–6.0) mmol/L.

**Table 1:** Baseline characteristics of the study population (*n* = 1740). Values are presented as mean ± s.d. and median (interquartile ranges) for continuous variables and percentages for dichotomous or categorical variables. P-values were derived by Fisher’s exact test or ANOVA.

Characteristics	Insulin			*P*-value	Total
	≤57	58–89	>90		
Number in category	587	615	538		1740
Age, years (s.d.)	74.0 ± 8.8	73.4 ± 9.1	71.1 ± 9.4	<0.001	72.9 ± 9.1
Women, %	48.9	47.2	41.4	0.03	46.0
Smoking, current, %	43.3	41.1	42.9	0.41	42.4
Diabetes mellitus, %	8.2	11.4	24.7	<0.001	14.4
Fasting glucose, mmol/L (s.d.)	5.3 (5.0–5.7)	5.6 (5.2–5.9)	5.9 (5.4–6.5)	<0.001	5.6 (5.2–6.0)
Fasting insulin, pmol/L	44 (35–51)	75 (66–85)	121 (100–165)	<0.001	74 (50–98)
Homa index	2.3 (2.1–2.5)	2.9 (2.7–3.1)	3.4 (3.2–3.8)	<0.001	2.9 (2.5–3.2)
Systolic blood pressure, mm/Hg (s.d.)	144 ± 20	147 ± 20	144 ± 20	0.02	145 ± 20
Diastolic blood pressure, mm/Hg (s.d.)	79 ± 10	81 ± 10	81 ± 11	0.001	80 ± 10
BMI, kg/m^2^ (s.d.)	25.7 ± 3.0	27.1 ± 3.1	29.2 ± 3.7	<0.001	27 ± 3.5
Waist circumference, cm	89.7 ± 9.9	93.8 ± 10.0	99.9 ± 11.0	<0.001	94.3 ± 11.1
Total cholesterol, mmol/L (s.d.)	5.6 ± 1.0	5.7 ± 1.0	5.5 ± 1.0	0.004	5.6 ± 1.0
HDL cholesterol, mmol/L (s.d.)	1.5 ± 0.3	1.4 ± 0.3	1.2 ± 0.3	<0.001	1.4 ± 0.3
Antihypertensive medication, %	32.0	36.6	50.4	<0.001	39.3
Antidiabetic medication, %	6.3	8.3	19.0	<0.001	10.9
Statin use, %	25.6	28.6	33.3	0.02	29.0
Vitamin K antagonists, %	5.8	5.7	5.2	0.90	5.6
Antiplatelet agents, %	27.6	26.5	28.4	0.76	27.5
Intima-media thickness, mm	3.2 ± 0.6	3.2 ± 0.6	3.2 ± 0.7	0.77	3.2 ± 0.6
Degree of stenosis, (%)	12.3 (0.0–25.9)	14.5 (0.0–26.4)	15.2 (0.0–28.0)	0.16	14.5 (0.0–26.8)
History of stroke, %	4.6	7.2	6.5	0.02	6.3
History of coronary heart disease, %	12.8	10.1	11.5	0.01	11.4
Presence of calcification, %	85.7	80.2	81.0	0.02	82.3
Presence of lipid core, %	47.2	45.5	38.8	0.01	44.0
Presence of intraplaque hemorrhage, %	32.5	35.9	35.5	0.41	34.7

Associations between fasting insulin and glucose levels with the different plaque components are summarized in Table 2. We found that higher fasting insulin levels were associated with the presence of intraplaque hemorrhage (fully adjusted odds ratio (OR) per 1-s.d. increase in insulin level: 1.38 (95% CI: 1.09–1.74)) (Table 2, model 2). Further adjustment for antithrombotic treatment increased the estimate and empowered the association (OR per 1-s.d. increase: 1.42 (95% CI: 1.12–1.79)) (Table 2, model 3). No association was found with the presence of calcification or lipid core. Also, when assessing the glucose levels, we found no association between fasting glucose levels with any of the plaque components. Furthermore, no association was found between the HOMA index and carotid plaque components (Table 2).

**Table 2:** Association serum insulin, glucose and HOMA index with carotid artery plaque composition (*n* = 1740) and glucose (per s.d. increment) with intraplaque hemorrhage (IPH), lipid core and calcification. Data are presented as OR (95% CI).

	IPH	Lipid core	Calcification
Insulin			
Model 1	1.27 (1.05–1.55)	0.76 (0.63–0.90)	0.93 (0.74–1.16)
Model 2*	1.38 (1.09–1.74)	0.89 (0.72–1.10)	1.06 (0.80–1.39)
Model 3	1.42 (1.12–1.79)	0.90 (0.73–1.11)	1.05 (0.79–1.39)
Glucose			
Model 1	1.04 (0.55–1.96)	0.51 (0.29–0.91)	1.19 (0.58–2.47)
Model 2^†^	0.47 (0.18–1.19)	1.09 (0.48–2.46)	0.90 (0.31–2.58)
Model 3	0.45 (0.18–1.16)	1.10 (0.49–2.49)	0.90 (0.31–2.57)
HOMA index			
Model 1	1.65 (1.01–2.71)	0.49 (0.32–0.77)	0.80 (0.45–1.44)
Model 2*^†^	0.37 (0.03–4.51)	1.33 (0.13–13.8)	0.05 (0.01–1.85)
Model 3	0.39 (0.03–4.74)	1.36 (0.13–14.1)	0.05 (0.01–2.01)

Data are presented as OR (95% CI). Model 1 = adjusted for sex, age, intima-media thickness, and the time difference between insulin and glucose measurements and MRI scan. Model 2 = model 1 + smoking, high-density lipoprotein, total cholesterol, systolic and diastolic blood pressure, diabetes mellitus, BMI, waist circumference, use of anti-diabetic medication, use of antihypertensive medication, and *glucose or ^†^insulin levels. Model 3 = model 2 + use of lipid-lowering medication, vitamin K antagonists, and antiplatelet agents.

**Figure 2:**
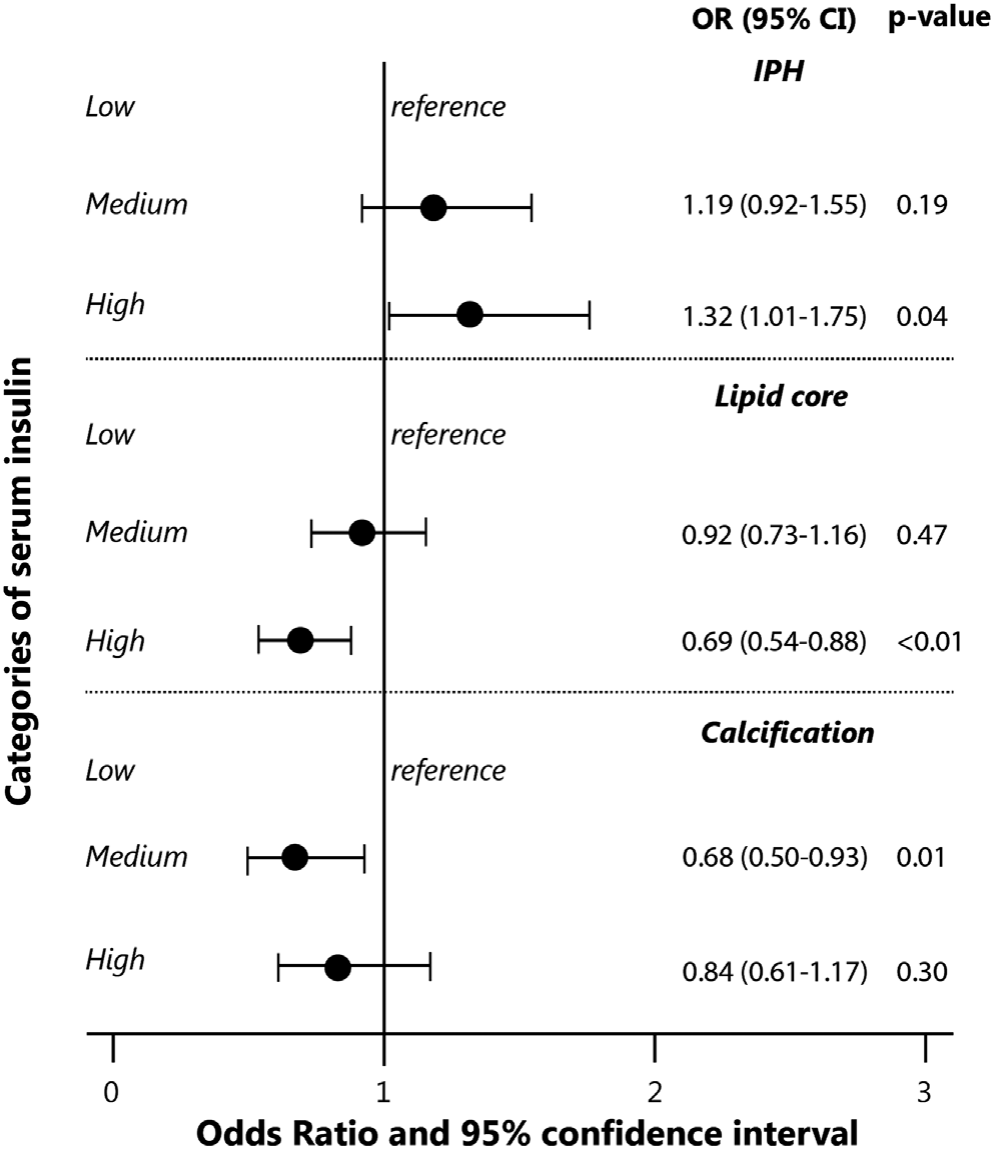
Association of serum insulin levels (tertiles) with plaque composition in the carotid artery. Values on the x-axis represent the odds ratios and 95% confidence interval. The values are adjusted for sex, age, and intima-media thickness. Statistics was performed using logistic regression using total sample of subjects *n* = 1740. *P-trend* over categories of insulin for intraplaque hemorrhage was 0.04, for lipid core was 0.003, and for calcification was 0.32.

**Figure 3:**
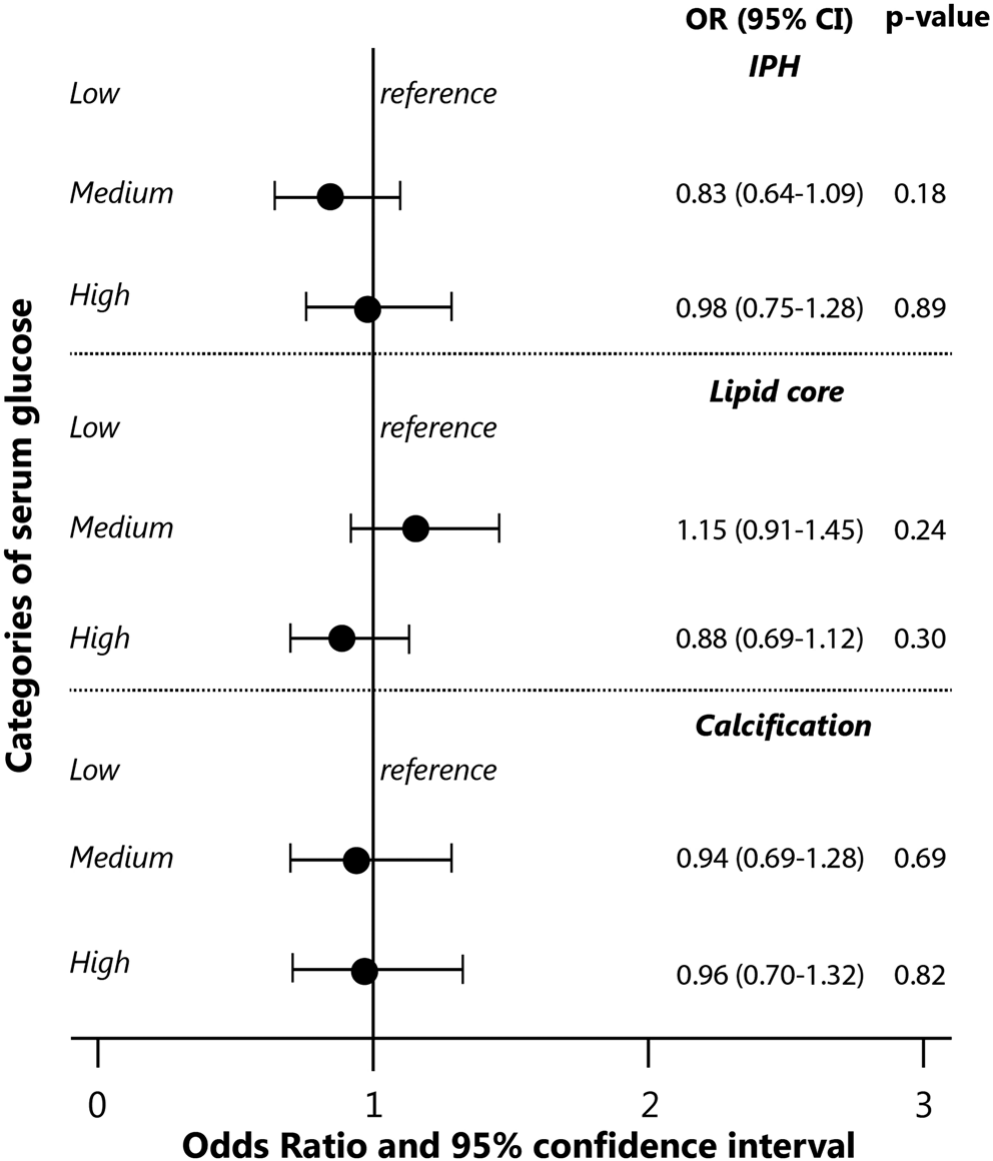
Association of serum glucose levels (tertiles) with plaque composition in the carotid artery. Values on the x-axis represent the odds ratios and 95% confidence interval. The values are adjusted for sex, age, and intima-media thickness. Statistics was performed using logistic regression using total sample of subjects *n* = 1740. *P-trend* over categories of glucose for intraplaque hemorrhage was 0.87, for lipid core was 0.33, and for calcification was 0.81.

When investigating tertiles of insulin and glucose levels, we found that the high insulin level tertile was associated with a higher frequency of intraplaque hemorrhage (adjusted OR of highest vs lowest tertile: 1.32 (95% CI: 1.01–1.75)) and a lower frequency of lipid core (adjusted OR of highest vs lowest tertile: 0.69 (95% CI: 0.54–0.88)) compared to the low tertile (Fig. 2). Again, for glucose, we did not find any association with the various plaque components (Fig. 3).

When restricting analyses only to participants who had both measurements (insulin and glucose measurements and MRI scan) within 6 months (*n* = 122) or less and within 1 year (*n* = 212), the results were in a similar trend (Supplementary Tables 1 and 2, see section on supplementary materials given at the end of this article). Similarly, the results did not change when we excluded participants with a diagnosis of diabetes mellitus at the time of MRI (*n* = 1489) (Supplementary Table 3). Furthermore, after stratifying the analyses by sex, similar effect estimates, between males and females, for the relation of serum insulin levels with IPH and lipid core were seen. Whereas no association was observed for calcification in both sexes. Also, when assessing the relationship between glucose and plaque components, no association was observed in either sex (Supplementary Table 4). Moreover, when assessing the relationship between serum insulin or glucose and carotid intima-media thickness, no association was found (Supplementary Table 5). No further stratified analyses were performed for the HOMA index.

## Discussion

In this large population-based study of subjects with subclinical carotid atherosclerosis, we observed that higher fasting serum insulin levels were associated with the presence of intraplaque hemorrhage within the carotid atherosclerotic plaque. We did not find an association between fasting glucose levels and any of the carotid plaque components.

Until now, most of the evidence linking insulin and glucose to atherosclerosis comes from studies in which atherosclerotic cardiovascular clinical endpoints, such as ischemic heart disease or ischemic stroke, were investigated (19, 20, 21). Hyperinsulinemia was found to increase the risk of ischemic heart disease among 4637 middle-aged men from the Quebec Cardiovascular Study (19) and the risk of acute coronary and cerebrovascular events in 1521 men enrolled in Kuopio Ischemic Heart Disease Risk Factor Study (20). Our results extend on these findings by showing that preclinical changes in serum insulin levels relate to a more vulnerable composition of the carotid atherosclerotic plaque. More specifically, we demonstrated that high serum insulin levels, especially related to the presence of intraplaque hemorrhage, the plaque component which is regarded as the most vulnerable (22). Similarly, insulin was found to increase intraretinal hemorrhage and extraretinal neovascularization in rats (23). Previous animal studies described insulin to play a pleiotropic effect on the vascular system, through vascular endothelial growth factor (VEGF), which plays a pivotal role in angiogenesis (23, 24). High levels of insulin increase the levels of VEGF, which in turn induce abnormal neovascularization that is prone to leakage and hemorrhage (23).

In contrast to high or low serum insulin levels, it may be speculated that physiological concentration levels (median levels 66–85 pmol/L) potentially behave protectively against atherosclerosis. Observations in our study showed that medium levels of serum insulin were not associated with any vulnerable plaque component, but were associated with a lower presence of calcification, which may support the hypothesis that medium levels of serum insulin have the protective effect (Fig. 2). In the same line, a recent animal study that examined the role of insulin in atherosclerotic plaque reported the protective effect of insulin on atherosclerosis (22). In this study, the insulin effect was tested on atherosclerosis in a mouse model, and insulin was found to decrease the plaque burden and increased plaque stability via nitric oxide synthase (NOS) mechanisms (22). Furthermore, it was found that insulin reduced macrophage accumulation and plaque necrosis and increased collagen and smooth muscle cell accumulation (22). However, it seems that only disrupted levels of serum insulin, low and high insulin levels, link serum insulin with atherosclerosis. Previously, animal studies demonstrated also that impaired insulin signaling by genetic modification accelerated atherosclerosis (25, 26, 27).

Surprisingly, we found no effects of serum glucose levels in carotid plaque composition. In the context of glucose, our findings are in contrast with studies that link glucose levels below 7.0 mmol/L with an increased risk of vascular disease (28). However, a recent meta-analysis of 102 prospective studies that investigated the relationship between fasting glucose levels and risk of vascular diseases concluded that glucose concentrations were non-linear and modestly associated with the risk of vascular diseases among individuals without diabetes (21), meaning that glucose levels below and higher than 7.0 mmol/L were associated with increased risk for coronary heart disease and ischemic stroke (21).

In terms of clinical practice, our findings may have clinical implications given that these suggest that fasting serum insulin conveys information on the atherosclerotic plaque composition that may ultimately be used for risk stratification of patients in daily practice.

The major strength of our study includes the largest population-based sample of individuals with subclinical carotid atherosclerosis and the MRI-based assessment carotid plaque composition. Given the accurate diabetes assessments within the Rotterdam Study, we were able to address, for the first time, the association between subclinical variations of insulin and glucose levels with atherosclerotic disease. Nevertheless, our study should be interpreted in the context of some limitations. First, the cross-sectional study design limits us to draw causal inferences between fasting insulin and atherosclerotic plaque components. Second, in a substantial part of our study population, the time interval between insulin and glucose measurements and MRI scanning was more than 2 years. However, limiting our analyses to the subgroup of participants with available measurements of MRI and serum insulin levels in the same year did show different associations.

## Conclusion

In conclusion, serum insulin levels are associated with the presence of vulnerable components of carotid plaque, specifically with intraplaque hemorrhage, suggesting that serum insulin may play a role in the vulnerability of carotid atherosclerotic plaque. Further studies are required to confirm our findings in a longitudinal design.

## Supplementary Material

Table S1 Association serum insulin and glucose levels with carotid artery plaque composition in the ≤ 6-months difference between MRI and insulin measurements (n=122)

Table S2 Association serum insulin and glucose levels with carotid artery plaque composition in the ≤ 1-year difference between MRI and insulin measurements (n=212)

Table S3 Association serum insulin and glucose levels with carotid artery plaque composition in individuals free of diabetes mellitus (n=1489)

Table S4 Association serum insulin and glucose levels with carotid artery plaque composition stratified by sex

Table S5 Association serum insulin and glucose levels with intima-media thickness (n=1740)

## Declaration of interest

The authors declare that there is no conflict of interest that could be perceived as prejudicing the impartiality of this study.

## Funding

The Rotterdam Study is supported by the Erasmus MC and Erasmus University Rotterdam; the Netherlands Organization for Scientific Research (NWO); the Netherlands Organization for Health Research and Development (ZonMw); the Research Institute for Diseases in the Elderly (RIDE); the Netherlands Genomics Initiative (NGI); the Ministry of Education, Culture and Science, the Ministry of Health, Welfare and Sports; the European Commission (DG XII); and the Municipality of Rotterdam. M K is supported by the VENI grant (91616079) from ZonMw. None of the funders had any role in the design and conduct of the study, collection, management, analysis, and interpretation of the data, and preparation, review, or approval of the manuscript.

## Author contribution statement

Study concept and design was performed by B M, D B, and O H F. Acquisition, analysis, or interpretation of data was performed by B M, D B, and O H F. Drafting of the manuscript was performed by B M. Critical revision of the manuscript for important intellectual content was performed by B M, D B, M K, A V L, J A S, O H F, and M W V. Statistical analysis was performed by B M. Administrative, technical, or material support was performed by B M, D B, and O H F. All authors read and approved the final manuscript. O H Franco and M W Vernooij contributed equally.
