# Supplementary material for: Serum insulin levels are associated with vulnerable plaque components in the carotid artery: the Rotterdam Study
Source: Eur J Endocrinol. 2020 Jan 20;182(3):343–50. doi: 10.1530/EJE-19-0620 (PMC7087499; doi:10.1530/EJE-19-0620)
Supplement: Table S1 Association serum insulin and glucose levels with carotid artery plaque composition in the ≤ 6-months difference between MRI and insulin measurements (n=122) [file supplementary_table_1.pdf]

Table S1 Association serum insulin and glucose levels with carotid artery plaque composition in the ≤ 6-months difference between MRI and insulin measurements (n=122)

Insulin	***IPH*** ***OR (95%CI)***	***Lipid core*** ***OR (95%CI)***	***Calcification*** ***OR (95%CI)***
Model 1	1.23 (0.65–2.32)	0.56 (0.29–1.11)	0.73 (0.33–1.59)
Model 2*	2.23 (0.88–5.66)	0.70 (0.27–1.77)	0.68 (0.17–2.64)
Model 3	2.41 (0.90–6.44)	0.64 (0.24–1.70)	0.44 (0.09–2.16)
Glucose			
Model 1	0.83 (0.12–5.82)	0.35 (0.05–2.46)	0.33 (0.02–4.03)
Model 2†	0.03 (0.01–1.13)	0.21 (0.07–5.80)	0.00 (0.00–0.70)
Model 3	0.04 (0.01–1.64)	0.24 (0.00–7.29)	0.00 (0.00–0.26)

Odds ratio (OR), given with a 95% confidence interval (CI), express the relationship between serum insulin and glucose (per SD increment) with intraplaque hemorrhage (IPH), lipid core and calcification. Model 1 = adjusted for sex, age, intima-media thickness and the time difference between insulin and glucose measurements and MRI scan. Model 2 = model 1 + smoking, high-density lipoprotein, total cholesterol, systolic and diastolic blood pressure, diabetes mellitus, body mass index, waist circumference, use of anti-diabetic medication, use of antihypertensive medication and *glucose or †insulin levels. Model 3 = model 2 + use of lipid-lowering medication, vitamin K antagonists and antiplatelet agents.
