# Supplementary material for: Serum insulin levels are associated with vulnerable plaque components in the carotid artery: the Rotterdam Study
Source: Eur J Endocrinol. 2020 Jan 20;182(3):343–50. doi: 10.1530/EJE-19-0620 (PMC7087499; doi:10.1530/EJE-19-0620)
Supplement: Table S2 Association serum insulin and glucose levels with carotid artery plaque composition in the ≤ 1-year difference between MRI and insulin measurements (n=212) [file supplementary_table_2.pdf]

Table S2 Association serum insulin and glucose levels with carotid artery plaque composition in the ≤ 1-year difference between MRI and insulin measurements (n=212)

Insulin	***IPH OR (95%CI)***	***Lipid core OR (95%CI)***	***Calcification OR (95%CI)***
Model 1	1.50 (0.85–2.64)	0.41 (0.23–0.74)	0.98 (0.55–1.75)
Model 2*	2.02 (0.97–4.19)	0.41 (0.20–0.84)	0.96 (0.45–2.06)
Model 3	2.41 (1.11–5.21)	0.40 (0.20–0.83)	0.97 (0.45–2.11)
Glucose			
Model 1	1.03 (0.20–5.44)	0.20 (0.36–1.08)	1.38 (0.25–7.58)
Model 2†	0.22 (0.20–2.84)	0.49 (0.05–4.88)	0.22 (0.02–2.64)
Model 3	0.16 (0.01–2.43)	0.52 (0.05–5.20)	0.21 (0.02–2.66)

Odds ratio (OR), given with a 95% confidence interval (CI), express the relationship between serum insulin and glucose (per SD increment) with intraplaque hemorrhage (IPH), lipid core and calcification. Model 1 = adjusted for sex, age, intima-media thickness and the time difference between insulin and glucose measurements and MRI scan. Model 2 = model 1 + smoking, high-density lipoprotein, total cholesterol, systolic and diastolic blood pressure, diabetes mellitus, body mass index, waist circumference, use of anti-diabetic medication, use of antihypertensive medication and *glucose or †insulin levels. Model 3 = model 2 + use of lipid-lowering medication, vitamin K antagonists and antiplatelet agents.
