# Supplementary material for: Serum insulin levels are associated with vulnerable plaque components in the carotid artery: the Rotterdam Study
Source: Eur J Endocrinol. 2020 Jan 20;182(3):343–50. doi: 10.1530/EJE-19-0620 (PMC7087499; doi:10.1530/EJE-19-0620)
Supplement: Table S3 Association serum insulin and glucose levels with carotid artery plaque composition in individuals free of diabetes mellitus (n=1489) [file supplementary_table_3.pdf]

Table S3 Association serum insulin and glucose levels with carotid artery plaque composition in individuals free of diabetes mellitus (n=1489)

Insulin	***IPH***<br>***OR (95%CI)***	***Lipid core***<br>***OR (95%CI)***	***Calcification***<br>***OR (95%CI)***
Model 1	1.32 (1.05–1.66)	0.78 (0.64–0.96)	0.99 (0.76–1.29)
Model 2*	1.43 (1.09–1.87)	0.87 (0.68–1.11)	1.08 (0.78–1.49)
Model 3	1.46 (1.12–1.92)	0.88 (0.69–1.12)	1.06 (0.77–1.47)
Glucose			
Model 1	0.71 (0.21–2.43)	0.90 (0.30–2.67)	1.31 (0.32–5.38)
Model 2†	0.40 (0.10–1.55)	1.85 (0.56–6.12)	0.91 (0.19–4.27)
Model 3	0.41 (0.11–1.60)	1.85 (0.56–6.18)	0.93 (0.20–4.38)

Odds ratio (OR), given with a 95% confidence interval (CI), express the relationship between serum insulin and glucose (per SD increment) with intraplaque hemorrhage (IPH), lipid core and calcification. Model 1 = adjusted for sex, age, intima-media thickness and the time difference between insulin and glucose measurements and MRI scan. Model 2 = model 1 + smoking, high-density lipoprotein, total cholesterol, systolic and diastolic blood pressure, body mass index, waist circumference, use of antihypertensive medication and *glucose or †insulin levels. Model 3 = model 2 + use of lipid-lowering medication, vitamin K antagonists and antiplatelet agents.
