# Supplementary material for: Serum insulin levels are associated with vulnerable plaque components in the carotid artery: the Rotterdam Study
Source: Eur J Endocrinol. 2020 Jan 20;182(3):343–50. doi: 10.1530/EJE-19-0620 (PMC7087499; doi:10.1530/EJE-19-0620)
Supplement: Table S4 Association serum insulin and glucose levels with carotid artery plaque composition stratified by sex [file supplementary_table_4.pdf]

**Table S4** Association serum insulin and glucose levels with carotid artery plaque composition stratified by sex

| **Females (n=800)** | | ***IPH*** ***OR (95%CI)*** | ***Lipid core*** ***OR (95%CI)*** | ***Calcification*** ***OR (95%CI)*** |
|---|---|---|---|---|
| ***Insulin*** | Model 1 | 1.16 (0.87–1.57) | 0.72 (0.55–0.94) | 0.95 (0.68–1.32) |
| | Model 2 | 1.25 (0.87–1.79) | 0.82 (0.60–1.13) | 1.04 (0.70–1.56) |
| | Model 3* | 1.26 (0.88–1.81) | 0.81 (0.58–1.12) | 1.02 (0.67–1.54) |
| ***Glucose*** | Model 1 | 1.80 (0.61–5.32) | 0.64 (0.24–1.72) | 1.15 (0.33–4.08) |
| | Model 2 | 1.13 (0.24–5.30) | 3.84 (0.92–16.13) | 2.27 (0.38–13.69) |
| | Model 3† | 1.11 (0.23–5.28) | 4.19 (0.98–17.88) | 2.42 (0.39–15.17) |
| **Males (n=940)** | | ***IPH*** ***OR (95%CI)*** | ***Lipid core*** ***OR (95%CI)*** | ***Calcification*** ***OR (95%CI)*** |
| ***Insulin*** | Model 1 | 1.35 (1.04–1.74) | 0.90 (0.68–1.17) | 0.92 (0.67–1.24) |
| | Model 2 | 1.52 (1.11–2.06) | 0.93 (0.70–1.22) | 1.08 (0.75–1.58) |
| | Model 3* | 1.57 (1.15–2.14) | 0.93 (0.71–1.24) | 1.07 (0.74–1.56) |
| ***Glucose*** | Model 1 | 0.74 (0.33–1.67) | 0.46 (0.22–0.95) | 1.18 (0.48–2.85) |
| | Model 2 | 0.31 (0.10–0.98) | 0.64 (0.24–1.71) | 0.85 (0.25–2.88) |
| | Model 3† | 0.30 (0.09–0.96) | 0.65 (0.24–1.72) | 0.84 (0.25–3.14) |

Odds ratio (OR), given with a 95% confidence interval (CI), express the relationship between serum insulin and glucose (per SD increment) with intraplaque hemorrhage (IPH), lipid core and calcification. Model 1 = adjusted for sex, age, intima-media thickness and the time difference between insulin and glucose measurements and MRI scan. Model 2 = model 1 + smoking, high-density lipoprotein, total cholesterol, systolic and diastolic blood pressure, diabetes mellitus, body mass index, waist circumference, use of anti-diabetic medication, use of antihypertensive medication and *glucose or †insulin levels. Model 3 = model 2 + use of lipid-lowering medication, vitamin K antagonists and antiplatelet agents.
