# Supplementary material for: Serum insulin levels are associated with vulnerable plaque components in the carotid artery: the Rotterdam Study
Source: Eur J Endocrinol. 2020 Jan 20;182(3):343–50. doi: 10.1530/EJE-19-0620 (PMC7087499; doi:10.1530/EJE-19-0620)
Supplement: Table S5 Association serum insulin and glucose levels with intima-media thickness (n=1740) [file supplementary_table_5.pdf]

Table S5 Association serum insulin and glucose levels with intima-media thickness (n=1740)

Insulin	***Beta***	***95% CI***	***p-value***
Model 1	0.009	-0.048–0.066	0.75
Model 2	-0.009	-0.077–0.059	0.80
Model 3*	0.001	-0.067–0.069	0.97
Glucose			
Model 1	0.034	-0.154–0.222	0.72
Model 2	0.015	-0.248–0.277	0.91
Model 3†	0.012	-0.249–0.274	0.93

Effect size, given with a 95% confidence interval (CI), express the relationship between serum insulin and glucose (per SD increment) intima-media thickness. Model 1 = adjusted for sex, age, intima-media thickness and time difference between insulin and glucose measurements and MRI scan. Model 2 = model 1 + smoking, high-density lipoprotein, total cholesterol, systolic and diastolic blood pressure, diabetes mellitus, body mass index, waist circumference, use of anti-diabetic medication, use of antihypertensive medication and *glucose or †insulin levels. Model 3 = model 2 + use of lipid-lowering medication, vitamin K antagonists and antiplatelet agents.
